# A SCOPING REVIEW OF SYSTEMS-LEVEL INTERVENTIONS FOR FALL PREVENTION IN LONG-TERM CARE

**DOI:** 10.1093/geroni/igad104.2597

**Published:** 2023-12-21

**Authors:** Andrew Ustach, Dahee Wi, Hilaire Thompson

**Affiliations:** University of Washington, Seattle, Washington, United States; University of Illinois Chicago, Chicago, Illinois, United States; University of Washington, Seattle, Washington, United States

## Abstract

Older adults residing in long-term care facilities (LTCFs) are at high risk for falls. In addition to individual resident-level interventions to prevent falls and fall-related injury (FRI), system-focused interventions are also necessary to adequately address fall prevention. Relatively little attention has been given to research involving systems-focused interventions. We aimed to synthesize studies on the effects of system-focused interventions for fall and FRI prevention in LTCFs with the goal of identifying promising strategies and gaps. We searched Medline, CINAHL, and EMBASE databases from 2017 to 2022 following the Preferred Reporting Items for Systematic reviews and Meta-Analyses extension for Scoping Reviews guideline. We conducted a narrative synthesis to summarize included studies. In the initial review, 301 studies were identified and underwent title and abstract screening resulting in 98 articles were retrieved for full-text review. Fifteen studies were included in data extraction. Fall prevention interventions evaluated in LTCF settings include: (1) multicomponent fall prevention programs, (2) staff education programs, (3) training in safe handling/transfers, (4) environmental adaptations, (5) electronic health record algorithms and prompts, (6) local culture change, (7) video analysis of falls to change local interventions, and (8) facility-level physical activity programs. About half of reported studies reported significant effects of system-focused interventions to reduce fall and FRI in LTCFs. Multicomponent fall prevention programs and physical activity interventions are the most effective systems-level interventions, while electronic health record interventions were the least effective. Overall, little attention has been given in the literature to evaluation of environmental adaptations at the systems level.

